# Stevens-Johnson Syndrome triggered by chemical hair relaxer: a case report

**DOI:** 10.4076/1757-1626-2-7748

**Published:** 2009-08-05

**Authors:** Matthew J Booker

**Affiliations:** School of Education, University of BirminghamEdgbaston, Birmingham, B15 2TTUK

## Abstract

This case report describes a 41-year-old Afro-Caribbean lady presenting with a constellation of pyrexia, conjunctivitis, arthralgia, sterile dysuria, apthous ulceration, labial crusting and widespread erythema multiforme. A diagnosis of Stevens-Johnson Syndrome was made. She had taken no medications recently (the most common precipitant of Stevens-Johnson Syndrome) and a full screen for the common and atypical bacterial and viral triggers was negative. The identified trigger was the use of a chemical hair relaxant treatment a few days previously. With supportive measures and a course of oral prednisolone, the patient quickly improved and made a full recovery. This case highlights the importance of considering occupational and recreational precipitants of Stevens-Johnson Syndrome.

## Case presentation

A 41-year-old Afro-Caribbean lady presented to A&E with a 72 hour history of sore red eyes, oral mucous ulceration, labial crusting, feverish arthralgia and widespread rash. She was normally fit and well, had no symptoms prior to the above, and normally worked as a retail assistant in a department store. She had no past medical history of note, took no regular medications or supplements, and had no recent travel abroad.

At presentation she was noticed to be febrile at 39°C, but otherwise haemodynamically stable. Examination revealed a pseudo-membranous conjunctivitis, extensive oral ulcerations and non-vesicular lip crusting with a muco-serous discharge. She had widespread ‘target lesions’ over her arms, legs and trunk, highly characteristic of erythema multiforme. Her urine analysis was normal, as was her chest x-ray and electrocardiogram. Her blood tests at presentation were largely normal, the only mild abnormalities being a slight lymphopoenia of 0.6 x 10^9^/L and a C-reactive protein level of 15 mg/L, rising to 191 mg/L some 24 hours later. Swabs of eyes and mouth gave no microbial growth, and no viruses were isolated by PCR. Her screens for varicella zoster virus, human immunodeficiency virus, herpes simplex virus and anti-streptolysin O were negative, as were blood cultures. Mycloplasma antibody levels were at an insignificant titre. A full genito-urinary screen was also negative.

A clinical diagnosis of Stevens-Johnson Syndrome was made, and she was commenced on a week-long course of 30 mg daily of prenisolone, and treated supportively with intravenous crystalloids, antipyretics, benzydamine hydrochloride mouthwash and empirical topical chloramphenicol to the eyes. An urgent ophthalmology opinion was obtained, revealing no other ocular manifestations. She improved quickly over the next few days, and was discharged home within a week.

Retrospectively, the only unusual contact the patient could recollect was having used a new chemical hair relaxation treatment 3 days prior to the symptoms arising. She recalls noticing some scalp irritation commencing a few hours after the treatment, but dismissed the significance of this initially.

In the absence of any infective or medication related precipitant, it seems possible that this chemical hair treatment was the trigger for the Stevens-Johnson Syndrome in this lady.

## Discussion

Stevens-Johnson Syndrome is classically a clinical diagnosis, thought to be due to a hypersensitivity complex affecting the skin and mucous-membranes. Current theories as to the immunological cause suggest a route involving CD8+ cytotoxic T-cells triggering keratinocyte apoptosis, but the current understanding is far from complete [1].

There are a host of well recognised medication triggers, particularly of note anti-epileptics, anti-retrovirals and many antibiotics [2]. Other common triggers are mycoplasma (and other atypical) pneumonias, and as a response to some viral infections. SJS can occasionally also be seen a result of acquired immunocompromise.

Despite approximately half of cases of Stevens-Johnson Syndrome (and the associated rarer more severe Toxic Epidermal Necrolysis) being idiopathic, there are some reports in the literature of chemical contacts inducing the syndrome, most frequently industrial chemicals (e.g. [3]) However, this is potentially the first report of a cosmetic hair product bringing about the phenomenon.

While this lady responded well to oral prednisolone, other treatment options for severe cases include cyclosporin and (more controversially) the use of intravenous immunoglobulin [4].

This case reports serves to highlight the importance of taking a detailed history in such presentations, and as a reminder that severe idiosyncratic reactions to widely available cosmetic and household products are an important cause of morbidity that should not be omitted from the differential.

## Abbreviation

PCR: polymerase chain reaction
